# Classification Predictive Model for Air Leak Detection in Endoworm Enteroscopy System

**DOI:** 10.3390/s22145211

**Published:** 2022-07-12

**Authors:** Roberto Zazo-Manzaneque, Vicente Pons-Beltrán, Ana Vidaurre, Alberto Santonja, Carlos Sánchez-Díaz

**Affiliations:** 1 Centre for Biomaterials and Tissue Engineering (CBIT), Universitat Politècnica de València, 46022 Valencia, Spain; vidaurre@fis.upv.es; 2 Digestive Endoscopy Unit, Digestive Diseases Department, La Fe Polytechnic Univesity Hospital, 46026 Valencia, Spain; pons_vicbel@gva.es; 3 Gastrointestinal Endoscopy Research Group, IIS Hospital La Fe, 46026 Valencia, Spain; 4 Biomedical Research Networking Center in Bioengineering, Biomaterials and Nanomedicine (CIBER-BBN), 28029 Madrid, Spain; 5 School of Design Engineering (ETSID), Universitat Politècnica de València, 46022 Valencia, Spain; asantonj@upvnet.upv.es; 6 Department of Electronic Engineering, Universitat Politècnica de València, 46022 Valencia, Spain; csanched@eln.upv.es

**Keywords:** classification predictive models, digital signal processing, enteroscopy, feature extraction, inflatable cavities, medical device, real-time detection system, soft robot

## Abstract

Current enteroscopy techniques present complications that are intended to be improved with the development of a new semi-automatic device called Endoworm. It consists of two different types of inflatable cavities. For its correct operation, it is essential to detect in real time if the inflatable cavities are malfunctioning (presence of air leakage). Two classification predictive models were obtained, one for each cavity typology, which must discern between the “*Right*” or “*Leak*” states. The cavity pressure signals were digitally processed, from which a set of features were extracted and selected. The predictive models were obtained from the features, and a prior classification of the signals between the two possible states was used as input to different supervised machine learning algorithms. The accuracy obtained from the classification predictive model for cavities of the *balloon-type* was 99.62%, while that of the *bellows-type* was 100%, representing an encouraging result. Once the models are validated with data generated in animal model tests and subsequently in exploratory clinical tests, their incorporation in the software device will ensure patient safety during small bowel exploration.

## 1. Introduction

Currently, there are different techniques for the exploration, diagnosis, and therapy of small bowel pathologies, the main one being enteroscopy. There are three different types of commercially available enteroscopes: single-balloon (SBE) [1], double-balloon (DBE) [2,3], and spiral (SE) [4]. Although these systems allow exploration of the small intestine, they are not without limitations [5,6,7,8,9].

In this context, a research group from the *Universitat Politècnica de València* and the *Fundación de Investigación del Hospital La Fe de Valencia* is working on the development of a new enteroscopy system, called Endoworm, which aims to improve the existing systems.

Endoworm is a semiautomatic soft robot device [10,11,12,13], which is mounted on a conventional endoscope and allows exploration of the small intestine. It consists of a pneumatic translation system of inflatable cavities governed by a microcontroller-based electronic device. The objective is to retract the intestine over the endoscope, thus assisting in advancing the endoscope [14,15].

The commercial systems used to explore the small bowel (especially DBE and SBE) have been tested for years, and some problems have been detected [16]; among them, no air leakage has been reported. The main reason for that is the manual operation of these two systems that allows the specialist to detect any malfunctioning of every inflatable cavity. Due to the automated operation of the Endoworm system and its complexity compared to the mentioned systems, the detection of malfunctioning in one of the three cavities is very difficult for the specialist, even more so to determine which one has failed. For this reason, developing an autonomous system for detecting air leakage from its inflatable cavities is necessary. An air leak in any of the cavities would represent a potential risk to the patient and even a significant loss in device efficiency to help the enteroscope advance through the small bowel.

Automated leak detection has been studied for different industrial applications. Leaks in rigid pipes have been investigated [17], some of them using machine learning methods [18]. In [19], a pneumatic system was analyzed to detect air leakage in the pipes and the pneumatic actuators while the system continues working. However, all the systems were rigid. Different methods to determine the leakage were presented in [20]. One of them used the pressure drop in the pipeline, again considering rigid pipes in industrial applications.

The complexity of detecting air leaks in Endoworm cavities lies in two aspects: their continuous inflation and deflation, which makes the static analysis of pressures ineffective, and the impossibility of seeing what is happening inside the patient, as well as the fact that it is not intended to introduce any electrical element that could cause damage. As described, leakage problems have been studied for industrial applications, but no previous studies have addressed this specific problem.

In medical applications, classification predictive models are commonly used to solve binary new cases classification, with high performance ratios [21]. These techniques can be used to classify the state of each cavity as “*Right*” or “*Leak*” while the device is working.

This work aimed to obtain two classification predictive models capable of detecting air leaks in the two different types of cavities (*balloon* and *bellows*) that make up the Endoworm translation system. The data used to generate the models were obtained by performing tests on the Endoworm enteroscopy system in in vitro models. The training and validation of the predictive models were carried out using *k-fold cross-validation* and test performance techniques. Finally, the best classification predictive model for each cavity type was selected, considering that they will have to be run in real time on the Endoworm control device.

## 2. Materials and Methods

Classification predictive models, which are intended to be obtained, require a series of input variables (features) that contain the necessary information on the pressure signals of the Endoworm cavities to classify the cases as “*Right*” or “*Leak*”.

The starting point to obtain the features was the differential pressure signals (relative to atmospheric pressure) received from the sensors arranged in the air outlet ports of the Endoworm control device. There are four signals in total: system pressure, pressure in the two radial expansion cavities (*balloons*), and the pressure in the axial expansion cavity (*bellows*). A detailed description of the Endoworm control device, the three inflatable cavities, and the inflation-deflating sequence can be found in [15].

The system pressure typically ranges from 200 to 300 kPa, depending on the configuration entered by the user. The pressure signals, measured in real time from the *balloons* and *bellows*, vary from 0 kPa to system pressure.

Of these four pressure signals, only the three corresponding to inflatable cavities are of interest for air leak detection. Therefore, these will serve as sources of information from which to extract the features to be used as inputs for the predictive model of each type of cavity (*balloons* or *bellows*).

The cavities are directly connected to the “*Honeywell*” pressure sensor “*26PCFFA2G*”, which can measure maximum differential pressure of 100 psi (689.476 kPa) with a sensitivity of 1 mV/psi (145.038 µV/kPa). This is important as the sensor model was changed from that used in [14,15], increasing the full scale of the measurement to 320 kPa. The gain of the AD620 instrumentation operational amplifier was readjusted to 107.73 V/V, achieving the desired full scale and a resolution of 0.313 kPa/bit. Between the output of the AD620 and the ADC input of the *PIC18F4550* microcontroller, an anti-aliasing filter (first-order passive low pass filter) was placed. The cutoff frequency of the anti-aliasing filter is 10 Hz. This frequency was selected because all the representative spectral content of the signals is contained below this cutting frequency. Moreover, it allows a reliable representation in the time domain of the fast transients present in the signals. The sampling frequency selected for the signals is 200 Hz, resulting in a time resolution of 5 ms per sample.

An external module was developed to acquire the main analogue and digital signals, in real time, that define the behavior of the control device (*Sniffer*), avoiding overloading the device’s control microcontroller.

**Figure 1:**
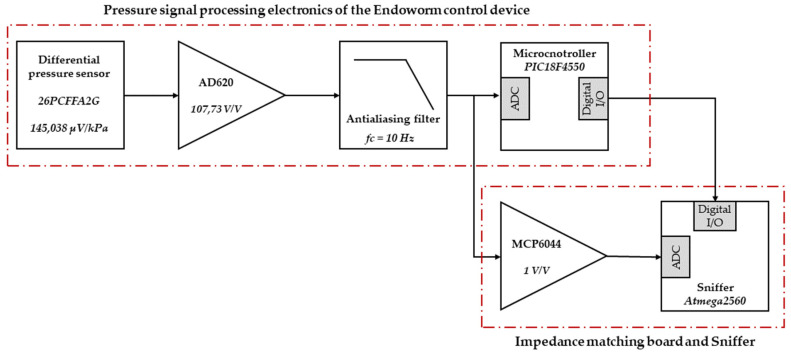
Diagram of the circuit for adapting the *26PCFFA2G* sensor output signal to the ADC input of the Endoworm microcontroller (*PIC18F4550*), as well as the capture of the pressure signals, through the impedance matching board, and the main digital control signals.

The *Sniffer* was a board based on the *Atmega2560* microcontroller. It was connected via a matching board to the microcontroller pins of the control device. The digital I/O of the *Sniffer* and the control device were connected directly. In contrast, the analogue signals from the pressure sensors were connected to the *Sniffer* via four *rail-to-rail* operational amplifiers in buffer configuration (*MCP6044)*, whose function was impedance matching between the inputs to the ADCs of the *PIC18F4550* and the *Atmega2560*. Figure 1 shows a block diagram illustrating the connection of the *Sniffer* to the control device.

The *Sniffer* records and sends to an external PC the pressure signals, the states of the solenoid valves, the state of the pneumatic pumps, and the main parameters of the *Finite State Machine*, which determine the control behavior of the device. The theoretical basis on which the programming of the Endoworm control device is based can be found in [22].

The data used to generate the predictive models were obtained by performing tests on the Endoworm enteroscopy system, aiming to capture the device’s normal functioning. A PU (polyurethane) artificial bowel model with an internal diameter of 40 mm was used for all tests. The intestine and the cavities used were characterized, and the results can be found in [14].

A total of 121 recordings were made, attempting to capture as much behavioral variability as possible in the operation of the Endoworm system. In some trials, the device was allowed to move freely (without being operated by any user); in others, it was driven by an expert endoscopist. The speed of the cavity sequence, its inflation–deflation times, and the system’s maximum pressure were varied. Three possible values for the sequence speed (“*Low*”, “*Medium*”, and “*High*”) could be established. The inflation and deflation times determined by the opening times of the solenoid valves varied from 0.050 to 0.125 s for *balloons* and from 0.8 to 1.4 s for *bellows*.

The “*Right*” or “*Leak*” operation was determined by the persons performing the experiment, on the basis of the observation of air leaks, for each inflation–deflating cycle of each cavity.

**Figure 2:**
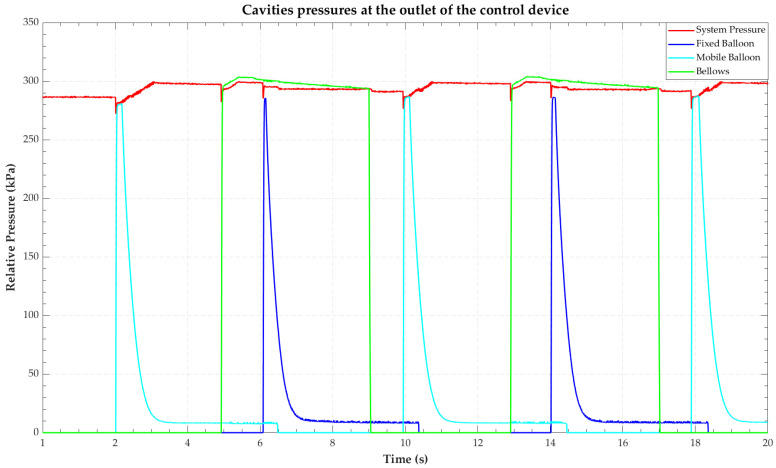
Temporal representation of the four pressures recorded by the *Sniffer* for any given test: system pressure (red), fixed balloon (dark blue), mobile balloon (blue), and bellows (green).

Figure 2 shows the pressure signals from the Endoworm control device corresponding to a segment of a recording in which all cavities were functioning correctly. 

The digital processing of the signals and the training of the classification predictive models were carried out using the “*Matlab R2022a*” software under a research license acquired by the *Universitat Poltècnica de València.*

A segmentation of the signals was carried out, consisting of two steps: signal swell detection and windowing of the signal. Swell detection was set as the instant at which the rising edge of the cavity pressure signal occurs. It was a robust parameter because whenever the solenoid valve was ordered to open, an abrupt rise in pressure was recorded in the sensors and calculated by detecting the positive peak of the derivative of the signal. From the time at which inflation occurs, a signal windowing was established to cover a complete cavity inflation–deflation cycle. This windowing was different depending on the type of cavity.

**Figure 3:**
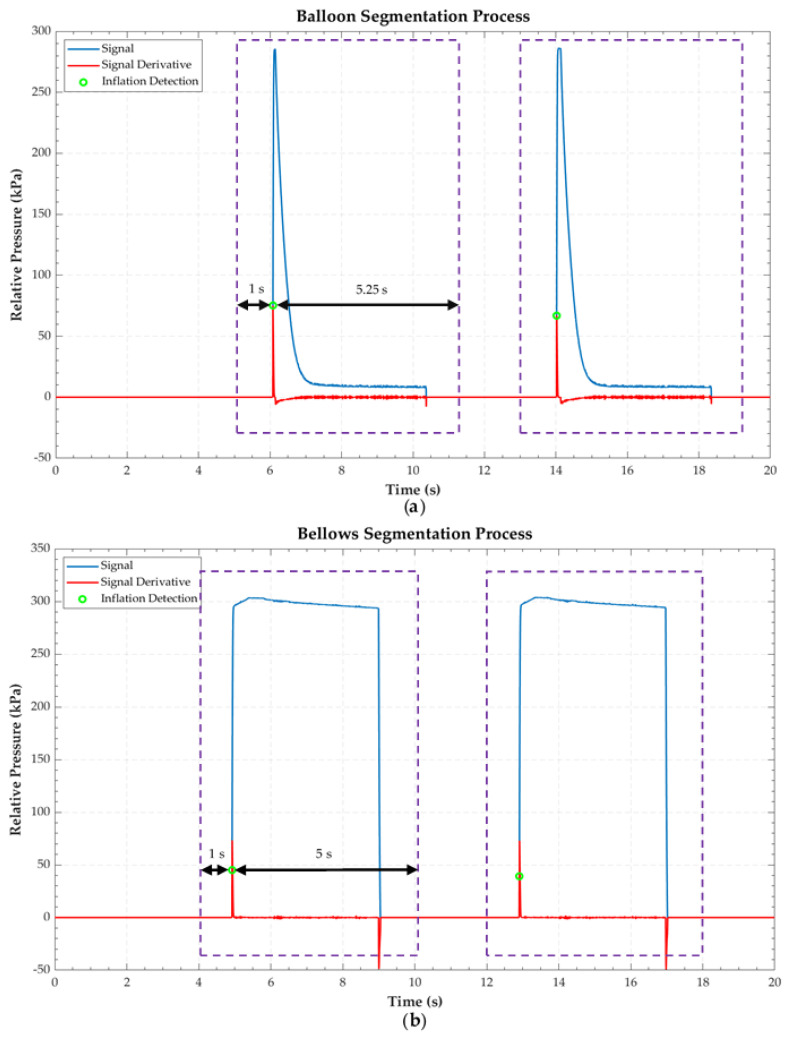
Graphical overview of the segmentation process of the two different Endoworm cavities: (**a**) *balloon-type* and (**b**) *bellows-type*. Dashed purple linear windows correspond to the segmented signal.

Figure 3 shows an example of the segmentation of each type of cavity, revealing the pressure signal, its derivative, and the instant in which the swelling of the cavity was detected. In addition, the time window corresponding to the segmented signal is shown.

**Figure 4:**
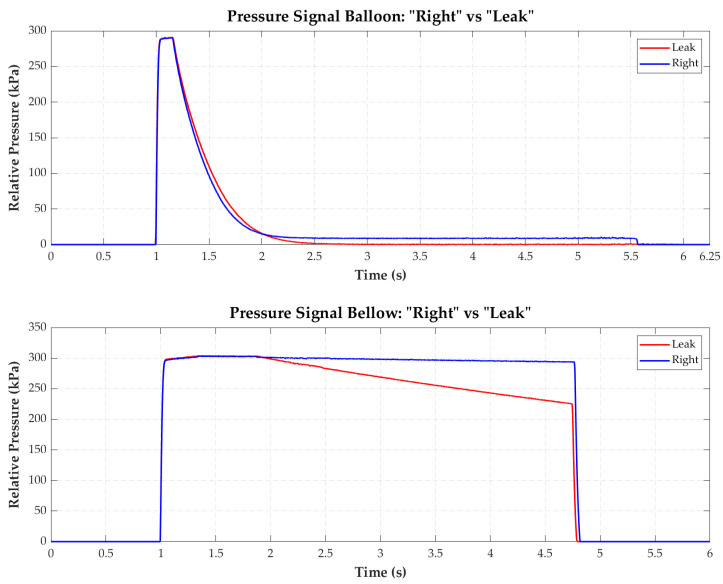
Representation of typical signals of the class *“Right”* (blue) and *“Leak”* (red) for the cavities *balloon* (**a**) and *bellows* (**b**).

A total of 2350 *balloon-type* segmented signals and 924 *bellows-type* segmented signals were obtained. These were assigned the label (“*Right*” or “*Leak*”) designated by the qualified personnel during the tests. Figure 4 shows typical signals representing the two possible states of the two cavity types that make up the Endoworm. 

Once the *balloon* and *bellows* signals were segmented and associated with their corresponding classification, they were randomized. The data for each cavity type were divided into two subsets: cross-validation (80%) and testing (20%).

Subsequently, the features of the signals were extracted. The features were identified to reflect, quantitatively or categorically, the physical–mathematical aspects of the signals to reflect the differences between cavities without and with leakage. In the case of the *balloon-type* cavities, a total of 12 features (*a*_1_–*a*_12_) were extracted, while, for the *bellows-type* cavities, a total of 13 features (*b*_1_–*b*_13_) were extracted. 

The features of both cavity types were divided into four subgroups: *a*_1_–*a*_3_, *a*_6_–*a*_8_, and *b*_1_–*b*_8_ to quantify pressure losses using different metrics; *a*_4_ and *b*_4_ to measure the time difference between the detection of inflation and deflation; *a*_10_, *a*_11_, and *b*_10_–*b*_12_ to indicate the correlation of the signal concerning different standard signals of the “*Right*” or “*Leak*” classes; *a*_5_, *a*_12_, and *b*_13_ to indicate categorical variables that, depending on a premise, assign a logical value (zero or one). Specifically, *a*_5_ indicates whether or not a deflating edge was detected, while *a*_12_ and *b*_13_ assign a label depending on the class of the pattern with the highest correlation value between the features *a*_10_ and *a*_11_ in the *balloon and b*_10_–*b*_12_ in the *bellows*, respectively. For a more extensive description of all extracted features, see Appendix A.

In order to optimize the performance of the algorithms and make their training more efficient, the descriptors were subjected to a normalization process using the *z-score* method [22,23,24].

The training of the predictive models was facilitated by a prior selection of features using *filters.* To increase the discriminant potential, those variables that obtained better results with respect to an objective function were selected. A total of four *filters* were used: *Fisher’s score* [25,26], *ReliefF* [27,28], *Chi-square* [29], and *MRMR* [30,31] (see Appendix B for a description of *filters*). Applying the four *filters*, four scores and ranking positions were obtained for each feature. A total score for the features was obtained by adding the ranking positions of each filter, which was lower for a better position. From this score, a total ranking was obtained that guarantees the same weight to the four *filters* when it comes to taking them into account when selecting the features. On this basis, the eight best characteristics were chosen for each type of cavity, discarding the rest.

Subsequently, the training and the validation of the predictive models were carried out. For this purpose, the *k-fold cross-validation* technique was used to make the most of the cross-validation subset, which was divided into *k-folds*. Typically, a low *k-value* means that the training subset was smaller and the validation subset was higher in percentage. This could result in a higher average prediction error (when averaging the results of the *k-folds*). In contrast, a high *k-value* would be the opposite; a higher training subset and a lower validation subset (in percent)could result in a lower average prediction error [32]. It is widely accepted to use a number of *k* between five and 20 folds [33]. A total of five folds were used for both types of cavities. This way, five instances of training were performed with 80% of the dataset for the *cross-validation* and 20% for validation. The ratio between the validation and test set was maintained at 20%.

The “*Classification Learner*” app in “*Matlab R2022a*” was used to obtain the classification predictive models. Algorithms that require a low computational cost and memory usage were selected because the models obtained were intended to be implemented in the device’s microcontroller. The following models (from the “*Classification Learner*” app) were trained: *fine, medium, and coarse tree*, *linear and quadratic discriminant*, *logistic regression*, *linear, quadratic and cubic SVM*, and *narrow, medium*, *and bilayered neural Network*, yielding a total of 12 different algorithms. For training the models, the default hyperparameter configuration of the app was maintained, and it was not modified during the whole training and validation process (*cross-validation*). It was not considered necessary to adjust the hyperparameters of each of the different algorithms.

During the cross-validation process, the wrapper technique was applied [34]. It started with the eight previously selected features, from which a reduced set that presented the best possible performance was obtained. The selected features were tested, and the test performance, together with the cross-validation, produced the final performance of each model. Due to high combinatoriality, the wrapper procedure gave rise to the training and validation of the 12 classification algorithms. Only the best-performing combinations of input features and algorithms are shown. The main metrics used for this purpose were accuracy, recall, precision, and F1-score [35]. Finally, the selected model of each type had a tradeoff among better performance, lower computational cost and memory usage, and a set of input features that were easy to compute.

## 3. Results

### 3.1. Structure of the Datasets

Table 1 and Table 2 show the prevalence of the two classes, “*Right*” and “*Leak*”, in the two available datasets, *balloon* and *bellows* cavities, respectively.

### 3.2. Preliminary Screening of Features

**Figure 5:**
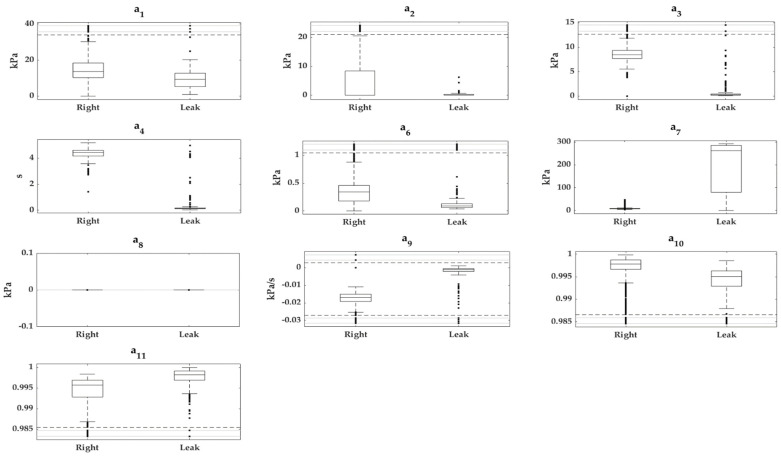
Box-and-whisker plots of quantitative features extracted (*a*_1_–*a*_4_ and *a*_6_–*a*_11_) from *balloon* signals. The “*Right*” and “*Leak*” groups are represented for each feature; *a*_5_ and *a*_12_ are not presented due to their categorical nature. For additional information on the extracted features see Appendix A.

**Figure 6:**
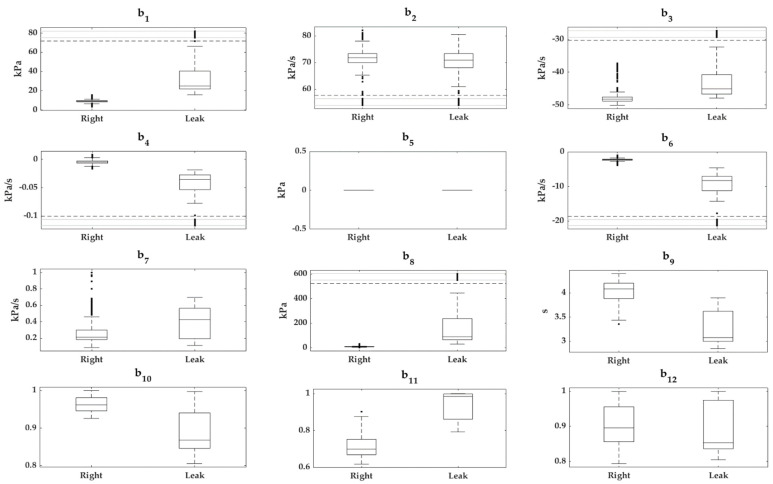
Box-and-whisker plots of quantitative features extracted (*b*_1_–*b*_12_) from the *bellows* signals. The “*Right*” and “*Leak*” groups are represented for each feature; *b*_13_ is not presented due to its categorical nature. For additional information on the extracted features see Appendix A.

Box-and-whisker plots of the quantitative features of the *cross-validation* subset for each cavity type are plotted in Figure 5 and Figure 6; categorical features were suppressed because they do not provide useful information in this type of plot. The cases were grouped according to whether they were previously classified as “*Right*” or “*Leak*”.

### 3.3. Feature Selection

Table 3 and Table 4 show the results of applying the filters for the *balloon* and *bellows* features, indicating scores and rankings by features.

### 3.4. Classification Predictive Models

This section presents a selection of the best results obtained by training the different classification predictive models and following the assumptions specified in Section 2. Table 5 shows the main results for leak detection in the *balloon-type* cavity, where the “*Leak*” class is defined as positive, and the “*Right*” class is defined as negative.

The best-performing and simplest classification predictive model for *balloon-type* cavities was the *medium tree* algorithm with input features *a_4_*, *a_6_* and *a_7_*. Its final performance was 99.62% *accuracy*, 98.11% *recall*, 99.45% *precision*, and 98.78% *F*1-*score*. 

**Figure 7:**
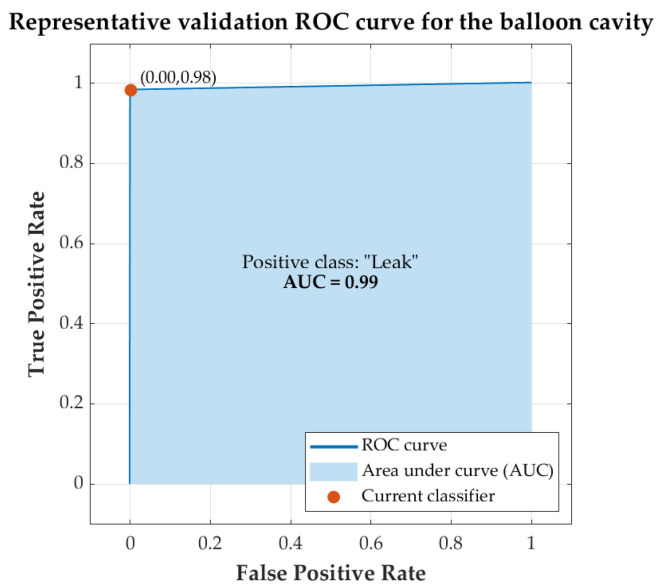
Representative validation ROC curve obtained for the *balloon* cavity model with *median tree* algorithm with input features *a*_4_, *a*_6_, and *a*_7_.

Figure 7 shows the ROC curve resulting from *cross-validation* for the classification predictive model obtained with *medium tree* algorithm with input features *a*_4_, *a*_6_, and *a*_7_. The ROC curve obtained for the best-performing *balloon* cavity models, shown in Table 5, is very similar to the one shown. The AUC of the best models of the *balloon* cavity was in the range of 0.97 to 0.99.

**Figure 8:**
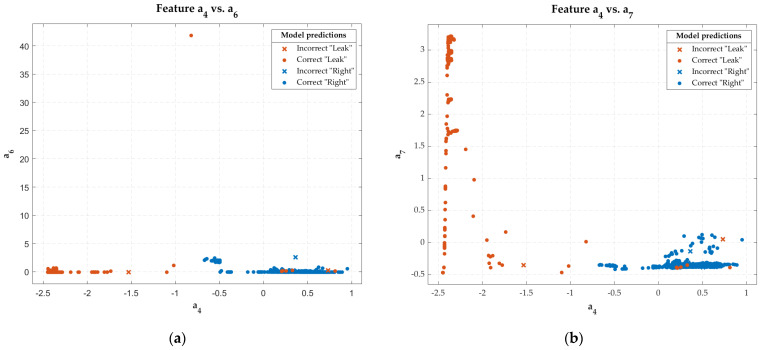

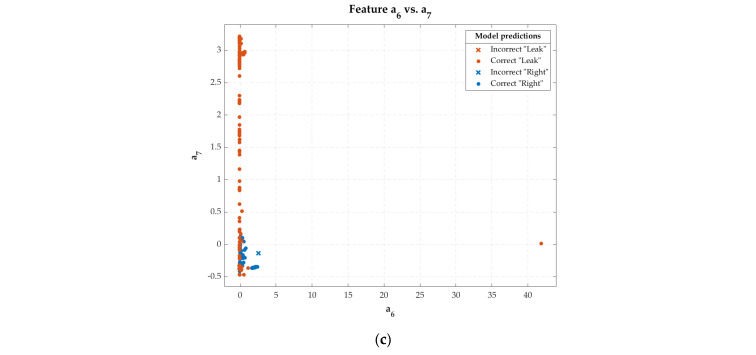
Scatter plots with the classification results of the predictive *medium tree* model with the input features *a*_4_, *a*_6_, and *a*_7_: scatter plot of feature *a*_4_ vs. *a*_6_ (**a**); scatter plot of feature *a*_4_ vs. *a*_7_ (**b**); scatter plot of feature *a*_6_ vs. *a*_7_ (**c**).

Figure 8 shows the scatter plots of the three input features to this predictive model. It also indicates the cases that were hit and miss in the model’s classification of the entire dataset.

On the other hand, the models of the *bellows* cavities obtained perfect results, with a correct classification for all the cases presented (100% *accuracy*). The best-performing algorithms were *logistic regression*, *linear SVM*, *neural networks*, and *coarse tree*, when features *b*_1_, *b*_4_, and *b*_6_ were part of the input set to the models.

**Figure 9:**
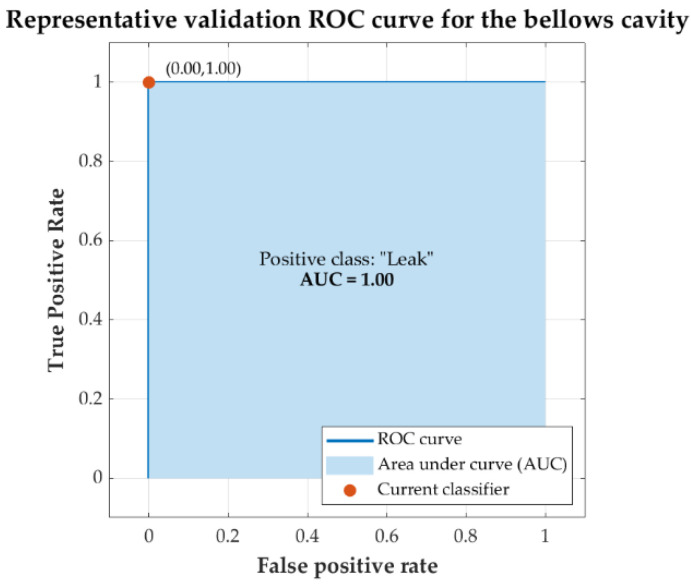
Representative validation ROC curve obtained for the *bellows* cavity model with *logistic regression* with input features *b*_1_, *b*_4_, and *b*_6_.

Figure 9 shows the ROC curve resulting from *cross-validation* for the classification predictive model obtained with the *logistic regression* algorithm with input features *b*_1_, *b*_4_, and *b*_6_. The ROC curve obtained for the best-performing *bellows* cavity models is identical to the one shown. The AUC was 1.00 for all models.

**Figure 10:**
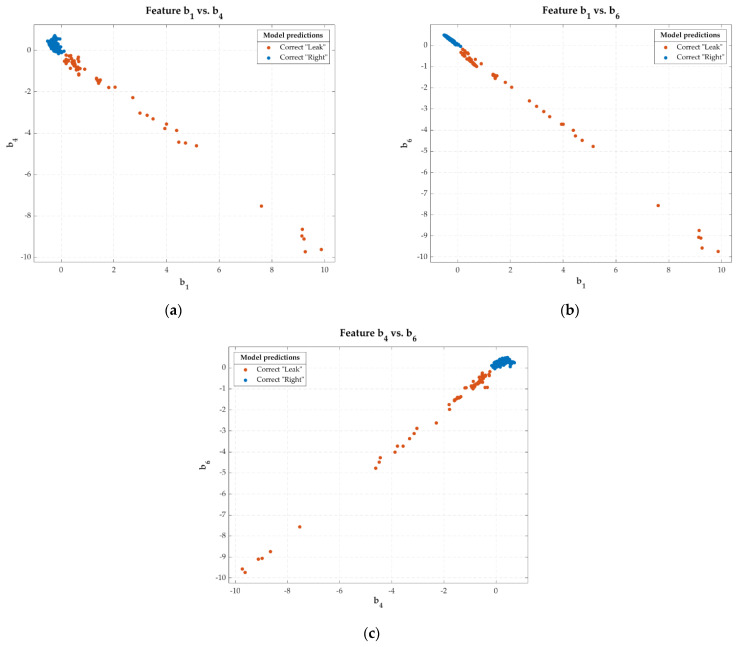
Scatter plots with the classification results of the predictive *logistic regression* model with the input features *b*_1_, *b*_4_, and *b*_6_: scatter plot of feature *b*_1_ vs. *b*_4_ (**a**); scatter plot of feature *b*_1_ vs. *b*_6_ (**b**); scatter plot of feature *b*_4_ vs. *b*_6_ (**c**).

Figure 10 shows the scatter plots of the three input features to this classification predictive model. It also indicates the cases that were hit and miss in the model’s classification of the entire dataset.

## 4. Discussion

The results showed an imbalance in the two datasets (*balloon* and *bellows)*, being around 85% for the “*Right*” class versus 15% for “*Leak*” for both (Table 1 and Table 2). This fact must be considered to evaluate the performance of the predictive classification. FN (false negative) mean that the device does not detect air leakage, resulting in unnecessary air injection into the patient’s small bowel, while FP (false positive) is the opposite case, resulting in unnecessary interruption of the scan due to false detections of leakage, which is also undesirable for the device’s proper functioning. In the issue at hand, it is preferable to give priority to FNs over FPs, as this prioritizes patient safety over the interruption of the scan. Therefore, the model with the best F1-score result and minimum number of FN was prioritized, i.e., the one with the highest *recall*. 

In the exploration of the cavity features, it was detected that *a_8_* and *b_5_* had a value of 0 kPa in all the samples (Figure 5 and Figure 6). This did not provide any discriminant capacity to our predictive models; hence, it can be anticipated that they will obtain very low scores when applying the *filters*. These two features belong to the subgroup of pressure parameters; more specifically, both indicate the pressure 0.5 s before cavity inflation occurs.

Figure 5 and Figure 6 show that the features *a*_1_, *a*_2_, *a*_6_, *a*_7_, *a*_10_, *a*_11_, *b*_2_, *b*_3_, *b*_7,_ and *b*_9_–*b*_12_ overlapped with the boxes and/or whiskers of the two classes, indicating that, on their own, they did not appear to have high discriminatory power between classes. In contrast, the features *a*_3_, *a*_4_, *a*_9_, *b*_1_, *b*_4_, *b*_6_, and *b*_8_ did not have overlapping boxes or whiskers between classes, but presented anomalous data that overlapped with the boxes and whiskers or anomalous data from the opposite class. The latter group of features had a high interclass discriminant potential on their own. 

Following the preliminary analysis of the features, the *filters* were applied; the results are shown in Table 3 and Table 4 for the *balloon* and the *bellows*, respectively. Calculating the total ranking, it was guaranteed that the four *filters* provided the exact weighting. On this basis, the eight best characteristics of each cavity type were selected, and the rest were discarded. Thus, *a*_1_, *a*_2_, *a*_8_, and *a*_12_ were discarded for *balloon-type* cavity models, and *b*_2_, *b*_3_, *b*_5_, *b*_7_, and *b*_12_ were discarded for bellows.

As mentioned in Section 3, the AUC ranges obtained in the training of the best models were between 0.97 and 0.99 for the *balloon* cavity models and 1.00 for the *bellows* cavity models. This indicates that the performances obtained by the models were excellent. Additionally, in the representative ROC curves of the models (see Figure 7 and Figure 9), it can be seen that the training of the models converged with few iterations, and that they were very close to the ideal ROC. 

Regarding the models obtained, it can be said that the algorithms that performed best from highest to lowest performance for *balloon* cavities were *medium tree*, *neural networks*, and *quadratic discriminant*. Furthermore, most of the interclass discriminating power, regardless of the algorithm, was concentrated in the features *a*_4_, *a*_6_, and *a*_7_ (see Table 5). It was observed that the combination of *a*_4_ with *a*_6_ and *a*_7_ gave high discriminatory power, as the cases of different classes tend to be grouped, except for some cases of “*Leak*” that were embedded within the cluster of cases of the “*Right*” class. However, *a*_4_ and *a*_7_ did not have such a clear separation of cases and tended to intermingle to a greater extent (see Figure 8).

On the other hand, the predictive models of the *bellows* cavities obtained perfect results, with a correct classification for all the cases presented. The algorithms with the best performance for this case were *logistic regression*, *linear SVM*, *neural networks*, and *coarse tree.* These excellent results could be explained by the absolute discriminant power of the *b_6_* feature, which was capable of 100% classification accuracy in the vast majority of algorithms. Excellent predictive power was obtained if this was combined with the *b_4_* feature (see Figure 10).

For the detection of air leaks in *balloon* cavities, we chose the classification predictive model obtained with the *medium tree* algorithm and the input features *a_4_*, *a_6_*, and *a_7_*. It had a final performance of 99.62% *accuracy*, 98.11% *recall*, 99.45% precision, and 98.78% *F*1-*score*. The model achieved the best result for the *F*1-*score/recall* ratio. Moreover, this model was chosen due to its low computational cost and memory usage, and the input features were simple to calculate.

Tree-type algorithms are implemented straightforwardly by nesting *“if–else”* statements that compare a given threshold of input features to the model (establishment of decision boundaries).

It had as input features *a*_4_, *a*_6_, and *a*_7,_ which were simple to calculate and required a low computational cost compared to other features. The feature *a*_4_ was the time in seconds from detecting balloon cavity inflation to detecting deflation. The feature *a*_6_ was the variability of the cavity pressure in kPa in the time span from when the pressure signal stabilized after the inflation transient to the detection of cavity deflation. The feature *a*_7_ was the average pressure in the time range between 100 and 50 ms before detecting deflation of the signal. 

The only features that were computationally and memory-intensive were those that belonged to or were directly related to the subgroup of features that showed the correlation of the signal to different standard signals of the “*Right*” or “*Leak*” classes (*a*_10_–*a*_12_ and *b*_10_–*b*_13_).

To detect air leaks in the *bellows* cavities, we chose the classification predictive model obtained with the *logistic regression* algorithm with the input features *b*_1_, *b*_4_, and *b*_6_, achieving a performance of 100% in all metrics. In addition to presenting unbeatable results, it was selected because a predictive *logistic regression* model is easy to implement on a microcontroller and requires a low computational cost. Moreover, the input features it used were easy to compute. The feature *b*_1_ was the difference (in kPa) between the pressure in the *bellow* cavity when inflation was detected and the pressure just before deflation was detected. The feature *b*_4_ was the average of the derivative of the pressure signal (kPa/s) at the instant between the detection of cavity inflation and the instant just before detection of cavity inflation. In contrast, feature *b*_6_ estimated the pressure slope (kPa/s) calculated as feature *b*_1_ divided by the time elapsed between the detection of cavity inflation and deflation. 

## 5. Conclusions

This article aimed to obtain two classification predictive models, one for each type of cavity (*balloon* and *bellows*), that detect the presence of air leaks from the cavity and that could be implemented within the Endoworm control device. The models served two purposes: safety for patients and effective functioning. The most important was patient safety against possible problems due to excess air insufflation in the small intestine after an air leak. The second was to provide the device with a mechanism to ensure its effective functioning. If any cavities leak air, the translation system will not effectively perform its function (i.e., to fix and retract the intestine over the endoscope advancing through the digestive tract).

In order to achieve this objective, a series of features were extracted after digital processing of the pressure signals. Features were analyzed and selected by filtering. Later, *fivefold cross-validation* with *wrappers* for the training and validation of different supervised classification predictive models was applied. Finally, these models were tested with a subsequent selection of the best set of features and algorithm that obtained the best results.

Following this procedure, it was concluded that the best model for air leakage detection in *balloon* cavities was crafted from the *medium tree* algorithm and the input features *a_4_*, *a_6_*, and *a_7_*. The best model for the bellows cavities was composed of the *logistic regression* algorithm and the input features *b*_1_, *b*_4_, and *b*_6_.

With regard to the features extracted from the signals of the *balloon* cavities, it can be concluded that the best discriminating results were obtained by combining that which measures the time the cavity remained inflated (*a*_4_) with two parameters that measured the pressure and variability of the pressure once the cavity inflation pressure stabilized (*a*_7_ and *a*_6_, respectively). On the other hand, the features extracted from the signals of the *bellows* cavities were those that measured the pressure loss while the cavity remained inflated, either incrementally (*b*_1_) or through the derivative or approximations thereof (*b*_4_ and *b*_5_, respectively).

The algorithm and features used in both models required relatively low computational costs, allowing them to be run on the microcontroller of the Endoworm control device without problems. This will allow detecting, practically in real time, the presence or absence of air leaks in the medical device during small bowel examinations in patients.

The results obtained in this article are encouraging and meet the proposed objectives, as models with a high degree of reliability were obtained. In the future, the models obtained in this article should be tested and validated with experimental data on animal models (pigs are generally used) and subsequently with clinical trials on patients.

## Figures and Tables

**Table 1:** Number of cases and percentage distribution of the classes for the subset of *balloon-type* data into which the initial dataset was subdivided.

Data Subset	Class	*N*	Percentage
**Cross-** **Validation**	Right	1593	84.73
	Leak	287	15.27
**Test**	Right	387	82.34
	Leak	83	17.66
**Total**	Right	1980	84.26
	Leak	370	15.74

**Table 2:** Number of cases and percentage distribution of the classes for the subset of *bellows-type* data into which the initial dataset was subdivided.

Data Subset	Class	*N*	Percentage
**Cross-** **Validation**	Right	637	86.20
	Leak	102	13.80
**Test**	Right	151	81.62
	Leak	34	18.38
**Total**	Right	788	85.28
	Leak	136	14.72

**Table 3:** Results of applying *filters* for the features of the *balloon* signals.

Features	Scores				Ranking
	Fisher’s	ReliefF	Chi^2^	MRMR	Total
***a*_1_**	0.218	0.123	114.2	1.5 × 10^−14^	10
***a*_2_**	0.386	0.000	160.3	2.9 × 10^−14^	11
***a*_3_**	2.610	0.049	708.1	2.8 × 10^−14^	5
***a*_4_**	15.824	0.073	711.9	3.9 × 10^−1^	1
***a*_5_**	12.875	0.000	65,535.0	9.5 × 10^−14^	2 or 3
***a*_6_**	0.002	0.054	576.7	5.9 × 10^−14^	8
***a*_7_**	2.032	0.017	630.8	1.1 × 10^−13^	4
***a*_8_**	0.004	−0.001	13.3	7.6 × 10^−14^	12
***a*_9_**	1.644	0.050	684.8	9.8 × 10^−14^	2 or 3
***a*_10_**	0.051	0.058	191.3	3.9 × 10^−14^	9
***a*_11_**	0.239	0.033	347.0	3.3 × 10^−1^	6
***a*_12_**	2.766	0.000	457.1	8.0 × 10^−14^	7

**Table 4:** Results of applying *filters* for the features of the *bellows* signals.

Features	Scores				Ranking
	Fisher’s	ReliefF	Chi^2^	MRMR	Total
***b*_1_**	0.442	0.043	278.627	0.098	5
***b*_2_**	0.017	0.000	2.338	0.002	12
***b*_3_**	0.433	0.010	99.692	0.099	9
***b*_4_**	0.570	0.034	278.873	0.199	2 or 3
***b*_5_**	0.000	0.000	0.000	0.000	13
***b*_6_**	0.579	0.042	278.873	0.131	2 or 3
***b*_7_**	0.283	−0.017	38.996	0.061	11
***b*_8_**	0.127	0.004	278.566	0.387	6 or 7
***b*_9_**	3.009	0.009	218.732	0.061	6 or 7
***b*_10_**	1.380	0.019	211.381	0.332	4
***b*_11_**	4.779	0.019	266.701	0.259	1
***b*_12_**	0.010	0.007	40.772	0.008	10
***b*_13_**	1.751	0.000	114.625	0.128	8

**Table 5:** The seven models that obtained the best results for leak detection in *balloon-type* cavities. Results are expressed as percentages.

Algoritm	Features	5-Fold Cross-Validation				Test				Total			
		Acc.	Recall	Pre.	F1-Sc.	Acc.	Recall	Pre.	F1-Sc.	Acc.	Recall	Pre.	F1-Sc.
Medium tree	*a_4_, a_6_, a_7_*	99.63	98.26	99.30	98.77	99.57	97.59	100	98.78	99.62	98.11	99.45	98.78
	*a_3_–a_7_, a_9_, a_11_, a_12_*	99.63	98.26	99.30	98.77	99.57	97.59	100	98.78	99.62	98.11	99.45	98.78
	*a_4_, a_6_*	99.36	97.91	97.91	97.91	99.79	98.80	100	99.39	99.45	98.11	98.37	98.24
Bilayered neural network	*a_3_–a_7_, a_9_, a_11_, a_12_*	99.41	97.21	98.94	98.07	99.57	97.59	100	98.78	99.45	97.30	99.17	98.23
Narrow neural network	*a_4_, a_6_, a_7_, a_9_*	99.36	97.21	98.59	97.89	99.79	98.80	100	99.39	99.45	97.57	98.90	98.23
Quadratic SVM	*a_4_–a_7_*	99.36	96.17	99.64	97.87	99.57	97.59	100	98.78	99.40	96.49	99.72	98.08
Medium neural network	*a_4_–a_7_*	99.36	98.26	97.58	97.92	99.36	96.39	100	98.16	99.36	97.84	98.10	97.97

## Data Availability

The data presented in this study are available on request from the corresponding author. The data are not publicly available due to the dataset is continually being expanded with new in vitro tests. In addition, it is expected that the data set will be completed in the future with in vivo tests in animals and later in humans, as mentioned in the conclusions of the paper. For this reason, if you would like an updated version of the dataset, please contact the authors directly.

## Appendix A

Bellow: the description of the features that were extracted from the pressure signals of the *balloon* (*a_i_*) and *bellows* (*b_i_*) type cavities are shown below.

***a*_1_:** Pressure drop occurring in the signal’s stationary regime while the cavity remains inflated. It is calculated as the difference between the pressure value 1.5 and 3 s after the detection of cavity inflation (kPa).

***a*_2_:** Pressure value close to the deflation of the signal. This is the pressure value 3 s after cavity inflation is detected (kPa).

***a*_3_:** Pressure in the stationary regime when the cavity is inflated. It is calculated as the average of sample pressures between 1.5 and 3 s after the detection of cavity inflation (kPa).

***a*_4_:** Time between detection of inflation and deflation of the cavity. It is calculated as the difference between the instant of time at which deflation is detected with inflation (s).

***a*_5_:** Categorical feature indicating whether cavity deflation is detected. Its value is ‘*1*′ if a negative peak in the derivative of the pressure signal is detected in the time interval between 1.5 and 4 s after the detection of cavity inflation; otherwise, its value is ‘*0*′ (dimensionless).

***a*_6_:** Variability of pressure in the stationary regime when the cavity remains inflated. It is calculated as the statistical variability of the pressure values between the time instants 1.5 and 3 s after the detection of cavity inflation (kPa).

***a*_7_:** Average pressure immediately before to the detection of cavity deflation. It is calculated as the average pressure between 100 and 50 ms before cavity deflation is detected (kPa).

***a*_8_:** Average pressure before the cavity is inflated. It is calculated as the average pressure of the values between 550 and 450 ms before cavity inflation is detected (kPa).

***a*_9_:** Average cavity deflation slope in the stationary regime when the cavity is inflated. It is calculated as the average of the derivative of the pressure values between 1.5 s after cavity inflation and one sample before signal deflation is detected. In the case where cavity deflation is not detected (feature *a*_5_), it is calculated as the average of values between 1.5 and 3 s after the detection of signal inflation (kPa/s).

***a*_10_:** Maximum correlation of the segmented pressure signal concerning four standard signals of the class “*Right*”. It is calculated by obtaining the correlation of the four standard signals concerning the segmented signal, assigning to the value of the feature the highest value of the four correlations obtained (dimensionless).

***a*_11_:** Maximum correlation of the segmented pressure signal concerning four standard signals of the class “*Leak*”. It is calculated using the same method as *a*_10_ (dimensionless).

***a*_12_:** Categorical feature indicating whether the signal is of the “*Right*” or “*Leak*” class on the basis of the correlation value of the signal concerning the standard signals of the two classes. It is assigned a value of ‘*0*′ if *a*_10_ is greater than *a*_11_ and ‘*1*′ otherwise (dimensionless).

***b*_1_:** Pressure loss that occurs while the cavity is inflated. It is calculated as the difference between the maximum pressure from the time cavity inflation is detected until 1 s later and the minimum pressure from the time cavity deflation is detected until 1 s earlier (kPa).

***b*_2_:** Maximum peak of the derivative of the segmented pressure signal, corresponding to cavity inflation. It is calculated as the maximum value of the derivative of the signal (kPa/s).

***b*_3_:** Minimum peak of the derivative of the segmented pressure signal, corresponding to cavity deflation. It is calculated as the minimum value of the derivative of the signal (kPa/s).

***b*_4_:** Average slope of the pressure loss that occurs while the cavity remains inflated. It is calculated as the average of the derivative values between the maximum peak (inflated) and the minimum peak (deflated) of the derivative of the pressure signal (kPa/s).

***b*_5_:** Average pressure before to cavity inflation. It is calculated as the average pressure between 200 and 250 ms before detection of cavity inflation (kPa).

***b*_6_:** Estimation of the pressure loss slope while the cavity remains inflated. It is calculated as the feature *b*_1_ divided by the time difference between the detection of deflation and inflation of the cavity (kPa/s).

***b*_7_:** Variability of the pressure loss slope while the cavity remains inflated. It is calculated as the statistical variability of the values of the pressure derivative between the maximum and minimum peak of the derivative (kPa/s).

***b*_8_:** Variability of pressure while the cavity remains inflated. It is calculated as the statistical variability of the pressure values between inflation and deflation detection (kPa).

***b*_9_:** Time the cavity remains inflated. It is calculated as the difference between the time instant at which deflation and inflation are detected (s).

***b*_10_:** Maximum correlation of the segmented pressure signal concerning three standard signals of the class “*Right*”. It is calculated by obtaining the correlation of the three standard signals with respect to the segmented signal, assigning to the value of the feature the highest value of the four correlations obtained (dimensionless).

***b*_11_:** Maximum correlation of the segmented pressure signal concerning two standard signals of the class “*Leak*”. It is calculated using the same method as *b*_10_ (dimensionless).

***b*_12_:** Maximum correlation of the segmented pressure signal concerning two standard signals of the class “*Leak*”. It is calculated using the same method as *b*_10_, but the standard signals are different from those used in *b*_11_ (dimensionless).

***b*_13_:** Categorical feature indicating whether the signal is of the “*Right*” or “*Leak*” class on the basis of the correlation value of the signal concerning the standard signals of the two classes. It is assigned a value of ‘*0*′ if *b*_10_ is greater than *b*_11_ or *b*_12_ and ‘*1*′ otherwise (dimensionless).

## Appendix B

The formulation of the four types of filters used for the selection of the features is shown below.

1. ***Fisher’s score*** was calculated according to Equation (A1).

(A1) $$F\left(k\right)=\frac{\sum_{i=1}^{c}n_{i}{\left(\mu_{k}^{i}-\mu_{k}\right)}^{2}}{\sum_{i=1}^{c}n_{i}{\left(\sigma_{k}^{i}\right)}^{2}}\;,$$

where $n_{i}$ is the number of the *i*-th class, $\mu_{k}^{i}$ and $\sigma_{k}^{i}$ are the mean and variance of the *k*-th feature in the *i*-th class, respectively, and $\mu_{k}$ is the mean of the *k*-th feature in all classes [26].

2. ***ReliefF score*** was calculated using the default algorithm in *Matlab 2022a* using the “*relieff*” function. This algorithm works best for estimating feature importance for distance-based supervised models that use pairwise distances between observations to predict the response. The theoretical development of the calculations made by the algorithm to obtain the feature scores was described in [27].
3. ***Chi*-*square score*** was calculated using the default algorithm in *Matlab 2022a* using the “*fscchi2*” function. This filter examines whether each predictor variable is independent of a response variable using individual *chi-square tests*, ranking the features using the *p*-values of the *chi*-*square* test statistics. The theoretical development of how the algorithm works to obtain the scores and the ranking was shown in [29].
4. ***MRMR score*** was calculated using the default algorithm in *Matlab 2022a* using the “*fscmrmr*” function. The MRMR algorithm is responsible for finding an optimal set of features that are mutually and maximally dissimilar and that can effectively represent the response variable. The algorithm’s goal is to minimize the redundancy of a set of features and maximize the relevance of a set of features to the response variable. The algorithm quantifies redundancy and relevance using the mutual information of the variables (by pairs of features) and mutual information between a feature and the response. The theoretical and mathematical development of the algorithm was shown in [31].
